# CT-like image based on 3D fast low-angle shot: superior diagnostic performance of ossification of the posterior longitudinal ligament

**DOI:** 10.1007/s00256-025-04891-9

**Published:** 2025-02-20

**Authors:** Sujin Kim, Guen Young Lee, Bo Mi Chung

**Affiliations:** 1 Department of Radiology, Chung-Ang University Hospital, Chung-Ang University College of Medicine, 102, Heukseok-Ro, Dongjak-Gu, Seoul, 156-755 Republic of Korea; 2 Department of Radiology, Chung-Ang University Gwangmyeong Hospital, 110, Deokan-Ro, Gwangmyeong-Si, Gyeonggi-Do, Republic of Korea

**Keywords:** Cervical vertebrae, Posterior longitudinal ligament, Ossification, Magnetic resonance imaging

## Abstract

**Objective:**

To evaluate the diagnostic performance of 3D fast low-angle shot (FLASH) compared with conventional MRI sequences for detecting OPLL.

**Materials and methods:**

This retrospective study included 106 patients who underwent cervical spine MRI and CT. Thirty-nine and 67 patients were enrolled in the OPLL and control groups, respectively. Diagnostic performance and reader confidence for detecting OPLL were compared between conventional MRI using turbo spin echo (TSE) and conventional MRI plus 3D FLASH. Interobserver agreement was also calculated. Three subgroups were defined within the OPLL group according to the sequences required for detecting OPLL (TSE group: cases that were diagnosed as OPLL by reviewing only TSE; 3D FLASH group: cases that were diagnosed by reviewing both TSE and 3D FLASH; none group: cases that were not diagnosed on MRI). The thickness of the OPLL was compared between the subgroups.

**Results:**

The diagnostic performance of both readers improved when 3D FLASH was added to conventional MRI, and the difference was statistically significant for reader 2 (*p* = 0.006). After adding 3D FLASH, reader confidence significantly increased (*p* < 0.001), and interobserver agreement improved from good to excellent. The three subgroups exhibited significantly different OPLL thicknesses (*p* = 0.008), with the thickest in the TSE group (4.5 mm), followed by the 3D FLASH (3.4 mm) and None groups (2.4 mm).

**Conclusion:**

3D FLASH can be helpful for detecting OPLL when combined with conventional T1- and T2-weighted imaging.

**Supplementary Information:**

The online version contains supplementary material available at 10.1007/s00256-025-04891-9.

## Introduction

Cervical ossification of the posterior longitudinal ligament (OPLL) is defined as ectopic bone formation within the posterior longitudinal ligament (PLL) [1, 2]. Cervical OPLL is a crucial diagnosis that can result in cord compression and myelopathy and increase the risk of spinal cord injury in traumatic events [1, 2]. The exact pathogenesis of OPLL remains unclear; however, both genetic and environmental factors are likely to contribute. The normal PLL, consisting of collagen and elastin fibers, is approximately 1–2 mm thick. In OPLL, fibroblastic hyperplasia and collagen overproduction lead to ligament hypertrophy, providing a scaffold for ectopic endochondral ossification, with subsequent mineralization and cartilage ingrowth forming mature bone [2, 3].

In suspected degenerative cervical spine diseases, cross-sectional imaging is important to localize the lesions and distinguish between disk herniations and osseous lesions such as OPLL or osteophytes [4]. MRI is a particularly useful for evaluating of soft tissue structures, including the spinal cord and nerves [5]. However, imaging bony cortex is challenging on MRI due to the short T2 relaxation time, which results in the absence of a signal on conventional MRI [6]. CT is superior for visualizing osseous structures and is the modality of choice for preoperative planning of OPLL because it accurately determines the ossified mass’ size and shape [5, 7]. However, CT has limitations, including radiation exposure and lower soft tissue contrast [4]. Therefore, both MRI and CT are commonly performed before surgery to evaluate soft tissue and osseous components of degenerative changes.

Recently, interest in CT-like MRI sequence has increased. Adding a CT-like contrast sequence to conventional MRI protocols may reduce the need for CT and streamline workflow. T1-weighted 3D gradient echo (GRE) sequences such as FLASH (fast low-angle shot) or VIBE (volumetric interpolated breath-hold sequence) are one of the CT-like MR techniques. In GRE sequences, a bright signal in the trabecular and cortical bone and a low signal in soft tissues can be acquired by subtracting all pixels in the image by the lowest mean value of the tissues surrounding the bone structures [6]. This approach has been applied to assess bony structures in various joints and bone tumors [6, 8–13], but studies on its use in cervical OPLL remain limited.

This study aims to compare the performances of 3D FLASH and conventional MRI sequences for OPLL diagnosis using CT as a reference standard.

## Materials and methods

### Patients

This retrospective study was approved by the institutional review board, and the requirement for informed patient consent was waived. Between May 2022 and December 2023, 695 adult patients (18 years or older) underwent cervical spine MRI in our institution. To be included in this study, patients were required to undergo both cervical spine MRI and CT within two months (*n* = 215). One radiologist reviewed MR images and medical records and patients were excluded according to the following criteria: (1) history of trauma at the cervical spine (n = 59); (2) previous surgery at the cervical spine (*n* = 34); (3) history of ankylosing spondylitis (*n* = 2); (4) metastasis or infection at the cervical spine (*n* = 7); (5) congenital anomaly (e.g., block vertebra) (*n* = 1); and (6) inadequate sequence (*n* = 6). Finally, 106 patients who underwent cervical spine MRI for symptoms related to degenerative spondylosis were enrolled in this study (64 males, 42 females; mean age, 64.3 years; SD, 12.3).

### Image acquisition

All cervical spine MRI scans were acquired using two 3-T scanners (Magnetom Skyra [n = 63] and Magnetom Vida [*n* = 43], Siemens Healthcare). Routine examinations at our institution included sagittal T2, sagittal T1, axial T2-weighted turbo spin echo (TSE), and sagittal T1-weighted 3D FLASH sequences. Axial and coronal reconstructions of T1-weighted 3D FLASH images were performed in all cases, with a slice thickness of 1 mm and no interslice gap. Gray-scale inversion was performed for each image to mimic the contrast in CT images. Detailed parameters are listed in Supplementary Table 1.

All cervical spine CT scans were acquired using 256-channel MDCT systems (iCT 256; Philips Medical Systems, Best, Netherlands; IQon-Spectral CT; Philips Medical Systems, Best, Netherlands). The imaging parameters were: 120 kVp tube voltage; 140 mA tube current; 0.4 s rotation time; 2.5 mm slice thickness; and 2.5 mm reconstruction interval. Sagittal and coronal reformatted images were created as follows: 2.5-mm slice thickness; no space between images; bone algorithm; window width 3000; and window level 730.

### Image analysis

A picture archiving and communication system (PACS) was used to review images. A senior radiologist reviewed the cervical spine CT scans of enrolled patients. The diagnosis of OPLL was based on CT findings when ossification was present at the PLL, posterior to the vertebral bodies, disks, or both. Thirty-nine patients were diagnosed with OPLL of the cervical spine (25 males, 14 females; mean age, 64.6 years; SD, 10.0) and 67 patients without OPLL were used as the control group (39 males, 28 females; mean age, 64.1 years; SD, 13.5). The OPLL types were classified as (1) continuous, flowing ossification at several levels and the intervening disks; (2) segmental, located posterior to each vertebral body and not extending beyond the adjacent disk level; (3) mixed, a combination of continuous and segmental types; and (4) focal, occurring at the disk level [2]. The presence of OPLL at each vertebral level from C2 to C7 was also recorded. The maximum thickness of the OPLL was measured on axial CT images.

MR analysis was performed independently by two radiologists with 9 and 15 years of experience in spinal imaging, respectively. They independently reviewed the MR scans and were blinded to the CT and plain radiography results during the two reading sessions. The first session consisted of an evaluation of routine cervical spine MR sequences with T1- and T2-weighted TSE. The second reading session was performed at least one month later. In the second session, the radiologists reviewed the MR examinations using 3D FLASH images in addition to T1- and T2-weighted TSE.

During both sessions, the presence and type of OPLL were recorded for each patient. In addition, OPLL occurrence at each vertebral level was documented. On TSE sequences, OPLL was recorded as present when T1- and T2-low-signal intensity bands of various thicknesses were observed between the bone marrow of the vertebral body and dural sac, with or without internal intermediate or increased signal intensity on T1-weighted images [14, 15]. On 3D FLASH, a high-signal-intensity band at the PLL was recorded as the OPLL. Readers graded their confidence on a four-point scale (0–49%; 0, 50–75% confidence; 1, 76–90% confidence; 2, > 90% confidence; (3). In the second session, the maximum OPLL thickness was measured using axial 3D FLASH.

### Statistical analyses

Descriptive statistics were reported for clinical characteristics of all included patients and CT features of patients with OPLL. Student’s *t*-test and chi-square test were applied to compare clinical features between the two groups. Diagnostic performance using either the TSE sequences alone, or the TSE sequences plus 3D FLASH was evaluated for each reading session. McNemar’s test was used to compare the performance results. The Wilcoxon signed-rank test was used to compare the diagnostic confidence from the two reading sessions. OPLL thicknesses measured using CT or 3D FLASH were compared by using a paired *t*-test. Interobserver agreement was evaluated using kappa statistics and intraclass correlation coefficients (ICC). Interobserver agreement was graded as poor (≤ 0.20), fair (0.21–0.40), moderate (0.41–0.60), good (0.61–0.80), or excellent (> 0.81).

Three subgroups within the OPLL group were defined as follows: (1) TSE group: cases that were diagnosed as OPLL by reviewing only TSE; (2) 3D FLASH group: cases that were diagnosed by reviewing both TSE and 3D FLASH; (3) and none: cases that were not diagnosed on MRI. Differences in OPLL thickness between the subgroups were evaluated using one-way ANOVA and post hoc analysis using Dunnett’s method. Statistical significance was set at *p* < 0.05. Analyses were performed using IBM SPSS 20 software (IBM Software Inc.). The post hoc power analysis was performed using G*Power software (version 3.1.9.7).

## Results

A total of 39 patients were included in the OPLL group (25 males and 14 females; mean age, 64.6 years) and 67 patients were included in the control group (39 males and 28 females; mean age, 64.1 years). The mean time interval between CT and MRI was 7.9 days for the OPLL group and 7.8 days for the control group. No significant differences were found between the two groups in age, sex, or time between the CT and MRI scans (*p* = 0.854, *p* = 0.550, and *p* = 0.980, respectively). Among the OPLL patients, myelopathy was present on MRI in 53.8% (21/39) of patients and 74.7% (29/39) underwent surgery. In the OPLL group, 636 vertebral levels were evaluated for the presence of OPLL and it was found at 92 levels. The segmental type of OPLL was the most common (*n* = 23, 59.0%), followed by mixed type (*n* = 12, 30.8%), localized type (*n* = 3, 7.7%), and continuous type (*n* = 1, 2.6%). The mean OPLL thickness measured by CT was 3.9 mm (range, 1.3–7.9 mm).

### Evaluation of the added value provided by 3D FLASH in OPLL diagnosis

Table 1 shows the diagnostic performances per patient and per level, for both readers in each reading session. The diagnostic performance for detecting OPLL improved for both readers after an additional review of the 3D FLASH images, and the difference was statistically significant for reader 2 (per patient: *p* = 0.006, per level: *p* < 0.001).

**Table 1:** Comparison of diagnostic performance between routine TSE sequences and TSE sequences with additional 3D FLASH in the diagnosis of OPLL

Type of imaging and statistics	Reader 1	Reader 2
Per patient (*n* = 106)		
First session (TSE)		
Sensitivity (%)	62 (46–75)	62 (46–75)
Specificity (%)	88 (78–94)	99 (92–100)
Accuracy (%)	78 (70–85)	85 (77–91)
Second session (TSE and 3D FLASH)		
Sensitivity (%)	74 (59–85)	85 (70–93)
Specificity (%)	97 (90–99)	97 (90–99)
Accuracy (%)	89 (81–93)	93 (86–96)
p values calculated using McNemar’s test	0.289	0.006*
Per level (*n* = 636)		
First session (TSE)		
Sensitivity (%)	53 (43–63)	54 (44–64)
Specificity (%)	97 (95–98)	99 (97–99)
Accuracy (%)	91 (88–93)	92 (90–94)
Second session (TSE and 3D FLASH)		
Sensitivity (%)	77 (68–85)	85 (76–91)
Specificity (%)	99 (98–100)	97 (95–98)
Accuracy (%)	96 (94–97)	95 (93–96)
*p* values calculated using McNemar’s test	0.127	< 0.001*

Numbers in the parenthesis are 95% confidence intervals. FLASH, fast low-angle shot; TSE, turbo spine echo; OPLL, ossification of the posterior longitudinal ligament

**Fig. 1:**
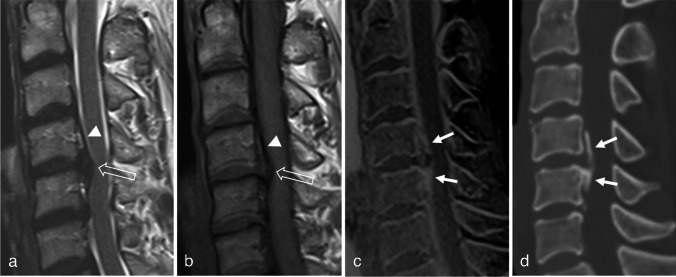
Fifty-three-year-old female who presented with posterior neck pain and radiating pain to right upper extremity. Sagittal T2-WI (**a**) and T1-WI (**b**) show disk herniation (open arrows) at C4/5 level. The 3D FLASH image (**c**) revealed the OPLL at C4,5 level (arrows). The sagittal CT image (**d**) confirms the diagnosis of OPLL (arrows). At the first reading session with sagittal TSE images, both readers could not diagnose the OPLL. The elevated thin hypointense structure (arrowheads in a and b) was thought to be the elevated posterior longitudinal ligament due to the herniated disk. After additional review of 3D FLASH image, both readers detected OPLL and corrected their decision

**Fig. 2:**
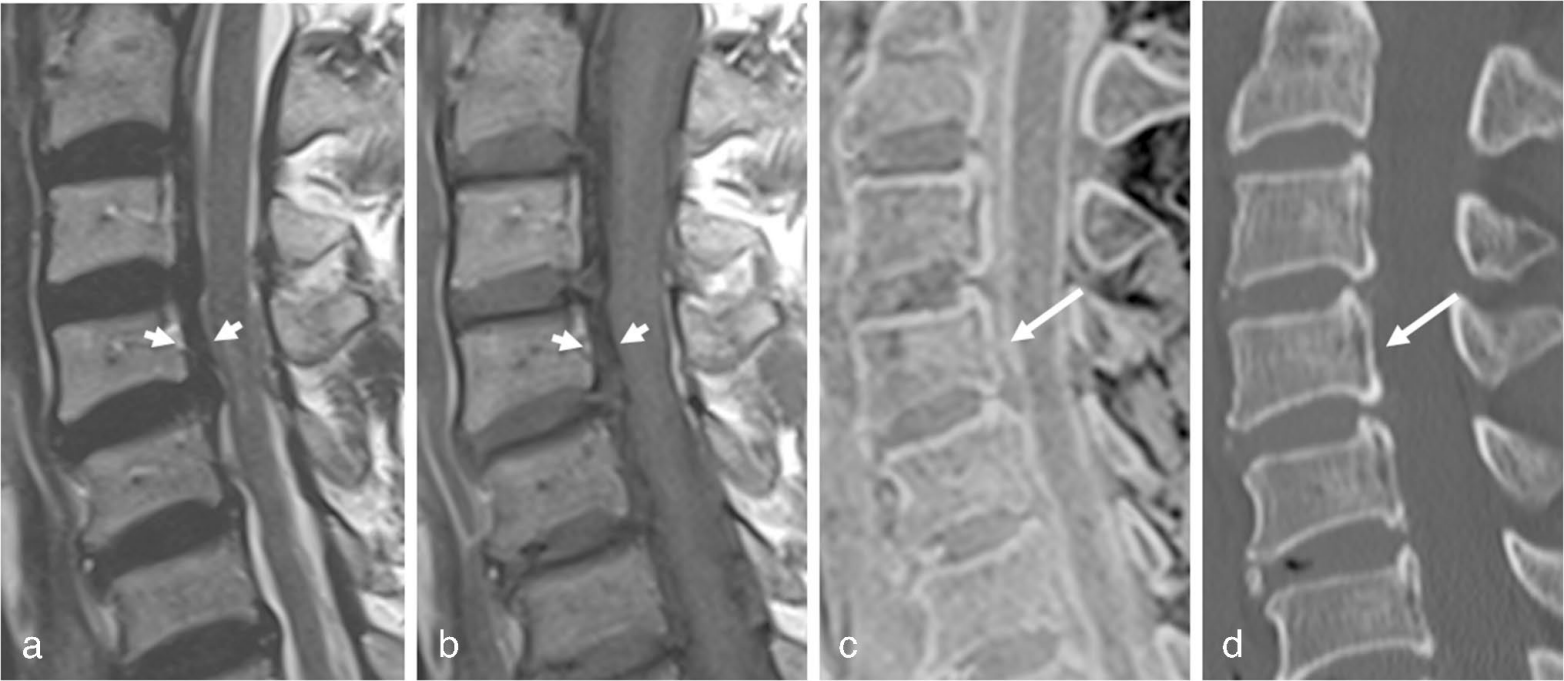
Eighty-two-year-old male who presented with weakness in both lower extremities. On sagittal T2- and T1-WI (**a** and **b**), a low-signal intensity band is seen between the posterior wall of the C4 vertebral body and dural sac (short arrows), mimicking calcified PLL. However, no OPLL was observed on 3D FLASH, which was confirmed by CT (arrows, **c** and **d**)

**Fig. 3:**
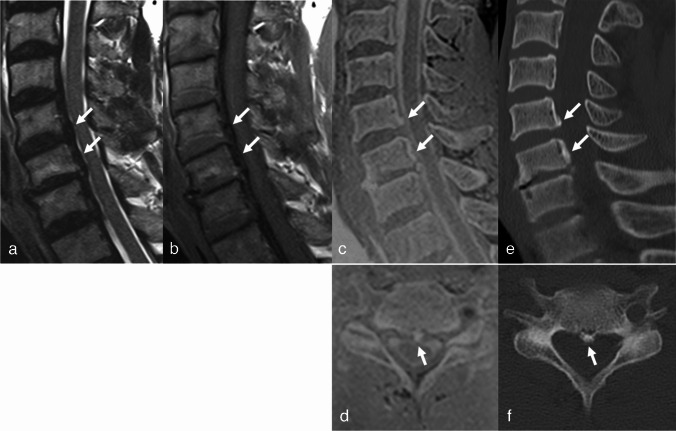
Fifty-eight-year-old male who presented with left upper extremity weakness. On sagittal T2- and T1-WI (**a** and **b**), a low-signal intensity band is seen between the posterior wall of the C5 and 6 vertebral bodies and dural sac (arrows). The sagittal 3D FLASH image (**c**) shows segmental OPLL at C5 and 6, which is confirmed on CT (**e**) (arrows). Note the OPLL at central zone of the C5 midbody on axial 3D FLASH (**d**) and CT images (**f**) (arrows). Both readers identified OPLL in the first and second reading, both increased their confidence grades from 1 to 3 after reviewing the additional 3D FLASH images in the second reading

Additional interpretation of the 3D FLASH images enabled the correct diagnosis of OPLL in 15, 10, and 6 cases for readers 1, 2, and both readers, respectively (Supplementary Table 2). Five OPLL cases were missed in the first session with TSE and were correctly diagnosed after a review of 3D FLASH by both readers (Fig. 1). One patient was over-diagnosed as OPLL on TSE by both readers, which was corrected after reviewing the 3D FLASH images (Fig. 2). The confidence grades in the second session with 3D FLASH were significantly higher than those in the first session for both readers (*p* < 0.001). Figure 3 shows an OPLL case where the confidence of both readers improved after review of 3D FLASH. Interobserver agreement for the presence and type of OPLL was good in the first session and excellent in the second session after an additional review of the 3D FLASH. Interobserver agreement was excellent on the thickness measurements (Table 2).

**Table 2:** Interobserver agreement

	κ	ICC	95% CI
Per patient			
Presence of OPLL			
TSE	0.642		0.479–0.805
TSE and 3D FLASH	0.824		0.708–0.941
Type			
TSE	0.636		0.483–0.788
TSE and 3D FLASH	0.861		0.761–0.961
Thickness		0.940	0.886–0.969
Per level			
Presence of OPLL			
TSE	0.750		0.661–0.838
TSE and 3D FLASH	0.833		0.770–0.896

*κ* kappa concordance, ICC intraclass correlation coefficient, *CI* confidence interval, *OPLL* ossification of the posterior longitudinal ligament, *TSE* turbo spin echo, *FLASH*, fast low-angle shot

### Subgroup analysis of OPLL thickness

**Fig. 4:**
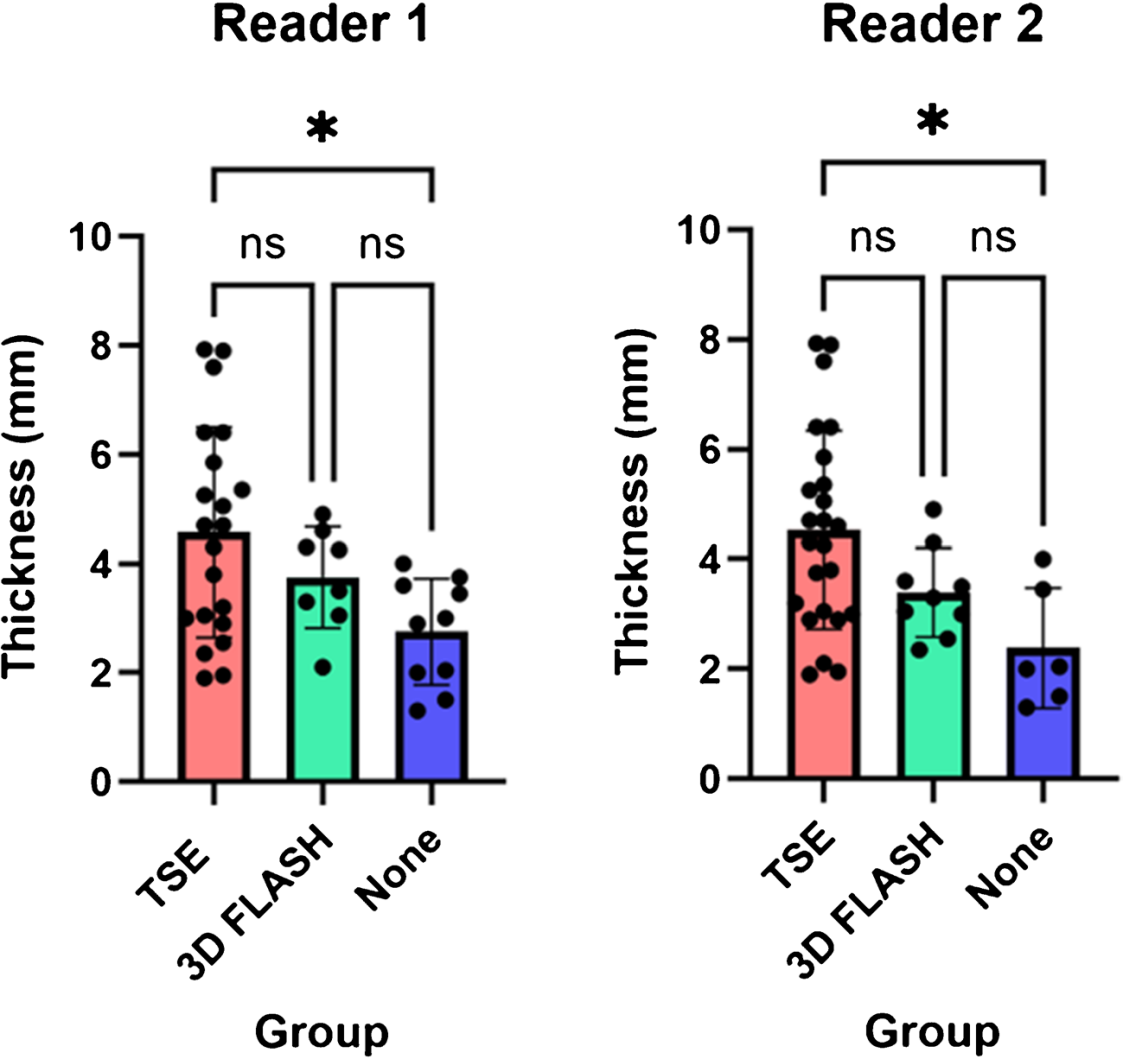
Results of one-way ANOVA analysis performed on the subgroups of the OPLL group. OPLL was thickest in the TSE group, followed by the 3D FLASH and none group in both readers. There were significant differences in OPLL thickness between the three groups. Specifically, post hoc analysis by both readers revealed significant differences between the TSE and none groups

The mean OPLL thicknesses of the three subgroups (TSE, 3D FLASH, and None) were 3.9 mm, 3.9 mm, and 3.8 mm on CT, for readers 1 and 2, respectively. We found no significant difference between the two readers in the thickness of OPLL measured on axial CT and 3D FLASH (CT vs. reader 1, *p* = 0.877; CT vs. reader 2, *p* = 0.364). Table 3 and Fig. 4 show the results of one-way ANOVA performed on the OPLL subgroups. The OPLL was thickest in the TSE group, followed by the 3D FLASH and None groups. Significant differences in OPLL thickness were observed among the three groups for both readers. Post hoc analysis revealed significant differences between the TSE and None groups for both readers. Although not significant, the *p*-value comparing the TSE and 3D FLASH groups for reader 2 was low (*p* = 0.053).

**Table 3:** Results of one-way ANOVA analysis between the subgroups of the OPLL group

	Reader 1	Reader 2
Group	Thickness (mean, 95% CI)	Thickness (mean, 95% CI)
TSE	4.6 (3.7–5.5), *n* = 21	4.5 (3.8–5.3), *n* = 24
3D FLASH	3.8 (3.0–4.5), *n* = 8	3.4 (2.8–4.0), *n* = 9
None	2.8 (2.1–3.5), *n* = 10	2.4 (1.2–3.5), *n* = 6
	*p* value	*p* value
One-way ANOVA	0.007*	0.008*
Post hoc analysis		
TSE vs. 3D FLASH	0.343	0.053
TSE vs. none	0.005*	0.008*
3D FLASH vs. none	0.119	0.220

Numbers in parentheses represent the 95% confidence intervals for the mean thickness. *n* indicates the number of patients. *p < 0.05. *OPLL*, ossification of the posterior longitudinal ligament, *CI* confidence interval, *FLASH* fast low-angle shot; *TSE*, turbo spin echo

## Discussion

This study demonstrated that adding 3D FLASH sequences enabled reliable detection of OPLL in cervical spine MRI with higher sensitivity and accuracy than that observed with conventional T1- and T2-weighted TSE sequences alone. Furthermore, reader confidence and interobserver agreement were better when 3D FLASH was added to the TSE sequences. The OPLL cases detected after reviewing 3D FLASH sequences tended to be thinner than those detected on TSE only.

Recent studies evaluated the osseous structures causing neural foraminal stenosis or central canal stenosis on cervical spine MRI using CT-like MR images [4, 16–18]. Most of these studies utilized ultrashort-echo time (UTE) or zero-echo time (ZTE) sequences, demonstrating the usefulness of these sequences for assessing bony components of neural foraminal stenosis or distinguishing disk herniation from posterior osteophytes [4, 16–18]. Schwaiger et al. compared two CT-like MR sequences, 3D GRE and UTE, for evaluating fractures and degenerative bone changes in the lumbar spine, using CT as a reference [19]. They reported that 3D GRE had higher agreement with CT and better image quality. UTE, while providing good tissue contrast, required twice the slice thickness for a similar acquisition time and was more sensitive to motion artifacts and B0 inhomogeneity, whereas GRE-based images were more affected by metallic artifacts. Based on their findings, 3D GRE was suggested to be more advantageous for clinical use under comparable imaging conditions.

Several reports have demonstrated the use of CT-like MR with ZTE and synthetic CT images in diagnosing OPLL [18, 20–22]. In a study by Tran et al., no significant difference was observed in the detection of OPLL with or without ZTE (OPLL was identified in 10 cases without ZTE and 11 cases with ZTE), which could be due to the small number of OPLL cases included [18]. Jeong et al. reported that reviewing synthetic CT increased sensitivity (reader 1: 47 to 90%, reader 2: 63 to 93%) but decreased specificity (reader 1: 98 to 89%, reader 2: 94 to 84%). In contrast, our study showed increases in both sensitivity and specificity, with a notable improvement in specificity. Similar to our findings, their study also reported improved inter-reader agreement, rising from moderate to good after adding CT-like images [22]. To the best of our knowledge, no study has investigated the utility of 3D T1 GRE-based CT-like images for detecting OPLL. In this study, using CT as a reference standard for all cases demonstrated that 3D FLASH could aid in reliable detection of OPLL.

Thick OPLL is well demonstrated on conventional MRI, with T1 intermediate to high SI in the OPLL reflecting fatty bone marrow. However, thin OPLL appearing as a thin hypointense band is difficult to distinguish from thickened PLL, disk herniation, or prominent anterior epidural vascularity [21]. Figure 1 shows a case of OPLL that was missed on the TSE. Both readers observed disk herniation, and a thin hypointense band was considered to indicate elevated PLL. After reviewing the 3D FLASH images, both readers detected OPLL which was confirmed by CT. In contrast, Fig. 2 illustrates an over-diagnosed case, where a low-signal intensity band posterior to the vertebral body on TSE imaging was mistaken for calcified PLL. This was disproven by 3D FLASH and CT images. The authors speculate that the hypointense band could be due to the PLL elevation from disk herniation and chemical shift artifact. Both cases highlight the difficulty of accurately assessing OPLL on TSE images and demonstrate the added value of 3D FLASH for clarification. Furthermore, considering the result that the OPLL in the TSE group was thicker than that in the 3D FLASH group, 3D FLASH appears to be especially helpful for difficult cases with thin OPLL. Reader confidence and interobserver agreement also improved after viewing the 3D FLASH. Therefore, 3D FLASH could serve as a problem-solving tool when an ambiguous osseous lesion is observed on TSE, thereby reducing the need for additional CT scans.

The implementation of CT-like MRI sequences in clinical practice could potentially streamline decision-making processes for cervical spine surgeries. Currently, many patients undergo both MRI and CT scans preoperatively. By incorporating these advanced MRI techniques, we may be able to simplify the diagnostic workflow, potentially reducing the need for separate CT scans in some cases. While further research is needed, this approach could lead to decreased healthcare costs, efficient resource utilization, and reduced radiation exposure for patients.

This study had several limitations. First, 3D GRE-based CT-like images are sensitive to susceptibility and motion artifacts. Although we excluded patients with a history of cervical spine surgery, 3D FLASH could provide inadequate image quality in such patients [19, 20]. However, this sequence is commonly available and has low hardware and software requirements [20]. Second, unfamiliar tissue signals can increase the difficulty of image interpretation when compared to CT. Therefore, experience with this particular sequence would likely be needed for accurate interpretation. Third, although posterior osteophytes are an important cause of central canal stenosis, we did not evaluate them because we intended to focus on OPLL. We plan to focus on posterior osteophytes in future studies. Fourth, this study used both per-patient and per-level analyses. OPLL-type classification required assessing overall morphology, necessitating per-patient analysis. For OPLL thickness, differences in angulation between CT and MRI made per-level measurements challenging, so we measured at the level with the greatest thickness and the most similar angulation between modalities. Although less detailed than per-level analysis, this approach addressed broader questions. Future studies with standardized imaging protocols could provide more detailed insights. Lastly, this retrospective design limited our ability to calculate the sample size prospectively. A post hoc power analysis using G*Power, however, confirmed that our sample size met the requirement for a power of 0.7 [23]. Furthermore, the statistically significant differences observed, in conjunction with the post hoc power analysis, suggest the sample size was adequate for the study objectives. Future studies are encouraged to aim for higher statistical power with sample sizes calculated during the planning phase for greater robustness.

In conclusion, 3D FLASH can be helpful for detecting OPLL when combined with conventional T1- and T2-weighted images. These results support increasing evidence that using CT-like MR to assess osseous structures can avoid further work-up with CT imaging.

## Supplementary Information

Below is the link to the electronic supplementary material.

Supplementary file1 (DOCX 19 KB)

## Data Availability

The data that support the findings of this study are available from the corresponding author, B. Chung, upon reasonable request.

## Abbreviations

OPLL: Ossification of the posterior longitudinal ligament
GRE: Gradient echo
FLASH: Fast low-angle shot
VIBE: Volumetric interpolated breath-hold sequence
TSE: Turbo spin echo
