# Supplementary material for: CT-like image based on 3D fast low-angle shot: superior diagnostic performance of ossification of the posterior longitudinal ligament
Source: Skeletal Radiol. 2025 Feb 20;54(9):1905–13. doi: 10.1007/s00256-025-04891-9 (PMC12241254; doi:10.1007/s00256-025-04891-9)
Supplement: Supplementary file 1 — Supplementary file1 (DOCX 19 KB) [file 256_2025_4891_MOESM1_ESM.docx]

Supplementary Table 1. MRI parameters for sagittal T1, sagittal and axial T2-TSE, and 3D FLASH sequences

|  | T1-TSE | T2-TSE | T2-TSE | 3D FLASH |
| --- | --- | --- | --- | --- |
| Orientation | Sagittal | Axial | Sagittal | Sagittal |
| TR (ms) | 651 | 2970 | 5230 | 25 |
| TE (ms) | 12 | 84 | 98 | 2.3 |
| FA (degrees) | 120 | 126 | 150 | 15 |
| FOV (mm) | 250 x 250 | 160 x 160 | 250 x 250 | 260 x 260 |
| Matrix | 384 x 288 | 384 x 269 | 256 x 280 | 288 x 288 |
| Voxel size (mm) | 0.3 x 0.3 x 2.0 | 0.3 x 0.3 x 3.0 | 0.3 x 0.3 x 2.0 | 0.5 x 0.5 x 0.5 |
| TA (min:s) | 3:28 | 3:40 | 2:53 | 4:40 |
| Slice thickness (mm) | 2.0 | 3.0 | 2.0 | 0.5 |
| Slice gap (mm) | 0.2 | 0.3 | 0.3 | 0 |
| Bandwidth (Hz) | 169 | 256 | 260 | 910 |

TSE, turbo spin-echo; FLASH, fast low-angle shot; TR, repetition time; TE, echo time; FA, flip angle; FOV, field of view; TA, acquisition time.

Supplementary Table 2. Cross-classification of results before and after 3D FLASH interpretation

| TSE 🡪 TSE and 3D FLASH | Reader 1 | Reader 2 | Both reader 1 and 2 |
| --- | --- | --- | --- |
| Correct 🡪 Correct |  |  |  |
| OPLL | 21 | 24 | 18 |
| Control | 58 | 64 | 56 |
| Incorrect 🡪 Correct |  |  |  |
| OPLL | 8 | 9 | 5 |
| Control | 7 | 1 | 1 |
| Correct 🡪 Incorrect |  |  |  |
| OPLL | 3 | 0 | 0 |
| Control | 1 | 2 | 0 |
| Incorrect 🡪 Incorrect |  |  |  |
| OPLL | 7 | 6 | 5 |
| Control | 1 | 0 | 0 |

Data are numbers of patients. FLASH, fast low-angle shot; OPLL, ossification of the posterior longitudinal ligament
